# Bioactive Glass-Based Endodontic Sealer as a Promising Root Canal Filling Material Without Semisolid Core Materials

**DOI:** 10.3390/ma12233967

**Published:** 2019-11-29

**Authors:** Ayako Washio, Takahiko Morotomi, Shinji Yoshii, Chiaki Kitamura

**Affiliations:** Division of Endodontics and Restorative Dentistry, Department of Oral Functions, Kyushu Dental University, Kitakyushu 803-8580, Japan; r05washio@fa.kyu-dent.ac.jp (A.W.); r13morotomi@fa.kyu-dent.ac.jp (T.M.); r08yoshii@fa.kyu-dent.ac.jp (S.Y.)

**Keywords:** bioceramics, bioactive glass, hydroxyapatite, root canal sealer

## Abstract

Endodontic treatment for a tooth with damaged dental pulp aims to both prevent and cure apical periodontitis. If the tooth is re-infected as a result of a poorly obturated root canal, periapical periodontitis may set-in due to invading bacteria. To both avoid any re-infection and improve the success rate of endodontic retreatment, a treated root canal should be three-dimensionally obturated with a biocompatible filling material. Recently, bioactive glass, one of the bioceramics, is focused on the research area of biocompatible biomaterials for endodontics. Root canal sealers derived from bioactive glass-based have been developed and applied in clinical endodontic treatments. However, at present, there is little evidence about the patient outcomes, sealing mechanism, sealing ability, and removability of the sealers. Herein, we have developed a bioactive glass-based root canal sealer and provided evidence concerning its physicochemical properties, biocompatibility, sealing ability, and removability. We also review the classification of bioceramics and characteristics of bioactive glass. Additionally, we describe the application of bioactive glass to facilitate the development of a new root canal sealer. Furthermore, this review shows the potential application of bioactive glass-based cement as a root canal filling material in the absence of semisolid core material.

## 1. Introduction

Endodontic treatment for a tooth with damaged dental pulp aims to both prevent and cure apical periodontitis. After a root canal preparation and irrigation in order to both remove bacteria and suppress the inflammation of the periodontal ligament around root apex, dentists obturate the treated root canal with filling materials [1]. If the post-treatment tooth is re-infected due to a poor root canal obturation, periapical periodontitis sets-in due to an invasion of bacteria into the canal. It is well known that the success rate of endodontic retreatment on periapical periodontitis is no higher than that of the initial treatment [2,3,4,5]. Three-dimensional obturation of the treated root canal with biocompatible filling materials is vital to avoid re-infection as well as the root canal preparation and irrigation steps, thereby increasing the success rate of retreatment [6,7]. 

The primary functions of any root canal filling material are to seal the bacterial in-growth so as to prevent fluid influx from providing nutrients to the trapped bacterium [8]. Endodontic treatment techniques have been changing due to technological advances, and advances in root canal filling material have significantly contributed to increased rates in the successful treatment of patients. Root canal sealer, one of the many filling materials, has been shown to be essential for successful obturation, as the sealer should bond to the dentin of the canal walls and close-off the periapical area of the root canal system. However, conventional root canal sealer typified by Grossman’s formula is hardly ideal as it is neither adhesive nor does it have a bonding effect with dentin. 

Mineral trioxide aggregate (MTA)-based root canal sealers, such as EndoSequence BC Sealer (Brasseler USA, Savannah, GA, USA), have been developed and are now commercially available; these MTA-based sealers provide ideal performance as a root canal sealer. MTA is formulated from commercial Portland cement (tricalcium silicate, dicalcium silicate, tricalcium aluminate, tetracalcium aluminoferrite, calcium sulfate) [9,10], combined with bismuth oxide powder for radiopacity. MTA-based root canal sealer is generally believed to be a bioceramic-based sealer. However, MTA is not bioceramic as its crystals are non-vitreous. It has been reported that some MTA-based sealers show good physical and biological properties [11,12,13], as well as the ability to produce hydroxyapatite on its surface in the presence of phosphate-buffered saline [14,15]. A hypothesized mechanism for the formation of hydroxyapatite is initiated by the release of calcium hydroxide from MTA, which interacts with a phosphate-containing solution to produce a calcium-deficient apatite achieved via an amorphous calcium phosphate phase [16]. These characteristics indicate that MTA-based sealers may display bioactivity. However, several studies report that some MTA-based root canal sealers show non-biocompatibility due to the presence of arsenic, a low ability to seal, long setting time, and non-retreatability [17,18].

Recently, bioactive glass, one of confirmed bioceramics, has been the focus of a great deal of research in biomaterials for Endodontics. Furthermore, bioactive glass-based root canal sealers have been developed and applied within clinical endodontic treatments. Herein we will review the appropriate classification of materials as bioceramics and the specific characteristics of bioactive glass. Additionally, we describe the possible application of bioactive glass as a newly developed root canal sealer is described. Furthermore, this review promises the potential of bioactive glass-based cement as a root canal filling material.

### 1.1. Bioceramics

Biomaterials are defined as synthetic or natural materials that are capable of either replacing parts of a living system or functioning while in intimate contact with living tissues [19]. Biomaterial-based implants and medical devices are widely used to replace or to restore the functionality of traumatized or degenerated tissues. The foremost requirement when selecting a biomaterial is its biological acceptability as a long-term non-rejected implant within the body. To achieve this acceptability, applicable biomaterials must be non-toxic, non-carcinogenic, chemically inert, stable, and mechanically strong. The most common biomaterial classes are metals, polymers, and ceramics. These three classes are used either solely or in combination to form the most presently available implantation devices.

Ceramics, a class of biomaterial, are polycrystalline materials that display characteristic hardness, brittleness, strength, stiffness, resistance to corrosion and wear, and low density. Bioceramics are utilized to restore functionality to diseased or damaged hard tissues and are used in several different fields such as dentistry, orthopedics, and medical sensors. Presently available bioceramics come in three basic types: bioinert, bioactive, and bioresorbable ceramics [20]. The first generation of bioceramics was comprised of alumina and zirconia [21]. The main features of first-generation bioceramics were their good mechanical properties, especially their wear resistance. The second generation of bioceramics was comprised of bioactive glass (BG), hydroxyapatite, and calcium phosphate-based cement. Second generation bioceramics bond to and integrate with the living bone of the body without forming a fibrous tissue around them and without promoting either inflammation or toxicity [22]. Unique among the second-generation bioceramics, BG has instigated a revolution in healthcare appliances and has paved the way for modern biomaterial-driven medicine [23,24].

### 1.2. Bioactive Glass 

**Figure 1:**
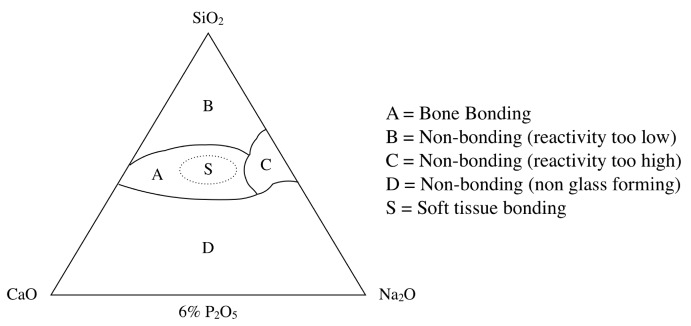
Compositional phase diagram of bioactive glasses with a focus on bone-bonding. Region S is the region of Class A bioactivity where bioactive glasses bond to both bone and soft tissues and display gene activating characteristics [25].

**Figure 2:**
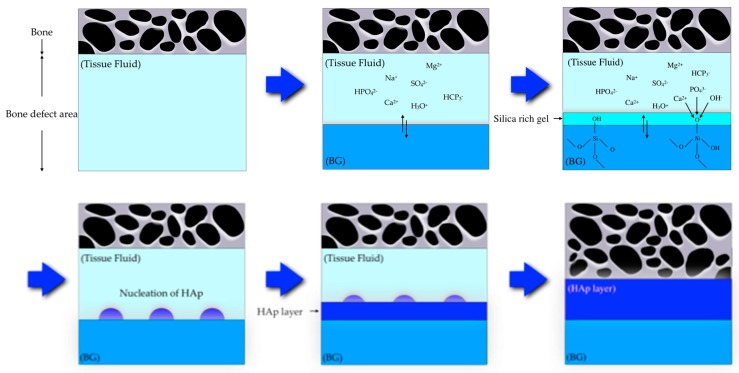
Scheme showing a proposed bonding mechanism of bioactive glass with bone [22,28].

BG contains the glass type of Na_2_O-CaO-SiO_2_-P_2_O_5_ in specific proportions [25], as a component of silica (SiO_2_) is ≤ 50 mol%. The compositional phase diagram for BG, also highlighting at what mixture-levels particular biomaterial properties arise, is provided as Figure 1 [25,26]. BG has been applied in clinical settings for orthopedic surgery for several decades. When BG is implanted in a defect area close to bone, reactions on BG surfaces lead to the release of critical concentrations of soluble Si, Ca, P and Na ions, which induce favorable intracellular and extracellular responses leading to rapid bone formation [27]; this bone formation is then followed by the formation of silica-rich gel on its surface. Silica-rich gel reacts with ions present in bodily fluids, resulting in the formation of hydroxyapatite (HAp)-like on the surface of BG. Furthermore, osteoblasts produce new bone in the silica-rich gel, allowing BG to bond with the bone through both the formation of bone-like hydroxyapatite layers and biological interactions with collagen (Figure 2) [22,28]. Additionally, BG is able to stimulate bone cells to regenerate and self-repair, thus significantly accelerating tissue healing kinetics [27]. These properties are termed osteoconductivity and osteoinductivity [23,29]. BG has been mainly used for applications where it will contact bone tissue, yet BG has recently shown promise in inducing the repair of soft tissues, too [30,31]. BG has attracted the interests of many researchers, as the ionic dissolution products of BG were found to stimulate angiogenesis. Furthermore, there now exist other BG-based products for applications in wound healing and peripheral nerve regeneration [32]. These applications suggest that BG shows suitability and biocompatibility as a biomaterial capable of being applied both to hard tissues such as dentin or cementum—as these materials are similar to the bone—and to soft tissues such as dental pulp and periapical tissue [33].

### 1.3. Bioceramic-Based Root Canal Sealer 

General practitioners desire a root canal sealer capable of strongly bonding to root canal walls with high sealing properties, high biocompatibility, as well as removability to accommodate retreatment. Researchers have found promising results in the application of bioceramics to solve these issues. Bioceramic-based materials have recently been introduced as endodontics materials as both repairing cement [34,35] and root canal sealer [13,36,37,38,39]. Bioceramic-based materials show an alkaline pH, antibacterial activity, radiopacity, biocompatible, nontoxic, non-shrinking, and are chemically stable within the biological environment. A further advantage of bioceramic materials is that they promote the formation of hydroxyapatite, ultimately facilitating a bond between dentin and the filling material during the setting process [11,38]. However, conventional bioceramic-based sealers show clinical disadvantages such as difficulty in handling, higher cytotoxicity in its freshly mixed state, a high pH during setting, long setting times, and that hardening requires sufficient moisture [18,40,41,42,43]. An additional disadvantage is that bioceramic-based sealers are difficult to remove when facilitating retreatment [44]. To overcome these disadvantages, we developed a next-generation bioceramic-based root canal sealer based on previous medically reliable BG-based materials, Nishika Canal Sealer BG (Nippon Shika Yakuhin, Yamaguchi, Japan).

### 1.4. Bioactive Glass-Based Root Canal Sealer

**Figure 3:**
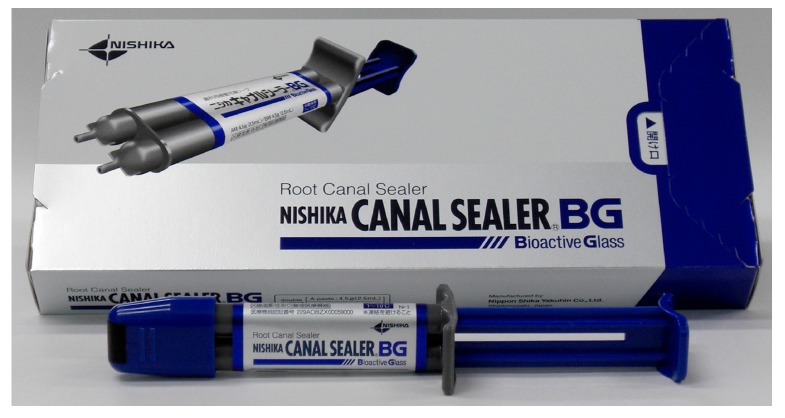
Nishika Canal Sealer BG.

**Figure 4:**
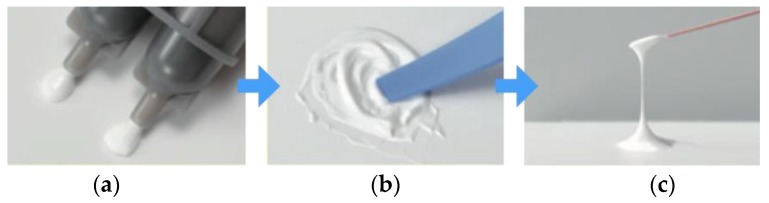
Mixing procedure of CS-BG. (**a**) Dispense the amount required. (**b**) Mix them gently. Mixing time: five seconds or more. (**c**) Ideal paste consistency for root canal obturation.

There are two well-known commercialized root canal sealers that include BG. One is GuttaFlow Bioseal (GFB) (Coltène/Whaledent AG, Altstätten, Switzerland), which is composed of gutta-percha, polydimethylsiloxane, platinum catalyzer, zirconium dioxide, and BG. GFB has shown a low solubility, low porosity, alkalization capacity [45], dentin penetrability [46], and cytocompatibility [47,48]. At present, only limited evidence is available concerning either the mechanism of GFB hardening or its ability to seal the canal and be removed for retreatment. The second product is Nishika Canal Sealer BG (CS-BG), shown in Figure 3; presently there exists compelling evidence concerning, with evidences about its physicochemical properties, biocompatibility, sealing ability, and removability. CS-BG was developed from BG-based biomaterials and originally intended for both dental pulp and bone regeneration therapies. CS-BG is a two-phased paste; Paste A consists of fatty acids, bismuth subcarbonate, and silica dioxide, whereas Paste B consists of magnesium oxide, calcium silicate glass (a type of BG), and silica dioxide, etc. By pushing the plunger of a double syringe, the two-phase paste can be dispensed at a 1:1 ratio. The dispensed paste can be mixed easily and quickly; this procedure is captured in Figure 4. A stainless-steel spatula may be corroded by the ingredients of the paste, we recommend the use of a plastic spatula to avoid contamination of metal implements. CS-BG paste tends to get hardened when exposed to heat or moisture. Therefore, it is recommended to store the syringes in the resealable aluminum foil bag, then placing the bag in a cold storage location (1–10 °C) without freezing. 

## 2. Physicochemical Properties

### 2.1. Physical Properties 

Physical properties of CS-BG were analyzed according to the International Organization for Standardization (ISO) standards of root canal sealing materials (ISO 6876:2012 Requirement), and it is found that the properties of CS-BG were suitable for use as an endodontic sealer (Table 1).

### 2.2. pH Change

**Figure 5:**
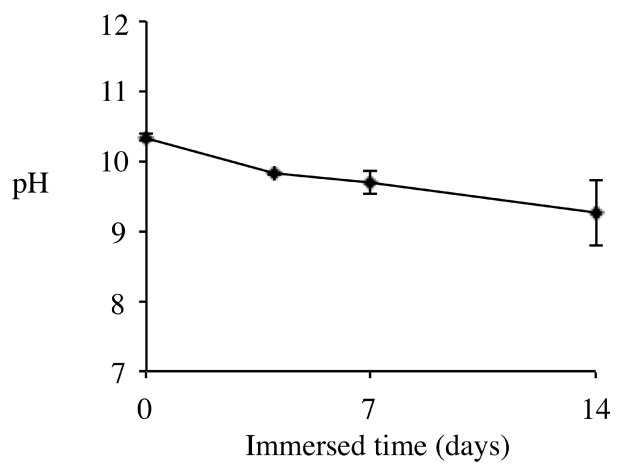
pH of purified water after immersion of hardened CS-BG [51].

The pH of a CS-BG sample that was hardened in simulated body fluid (SBF) was measured in the purified water. The pH gradually decreased during periodic immersion and stabilized at around pH = 10 (Figure 5); this pH is optimal for the formation of HAp on the BG surface [49,50,51]. This alkaline pH is maintained by ions evolving from non-BG components.

### 2.3. HAp Formation on the Surface of CS-BG in SBF

**Figure 6:**
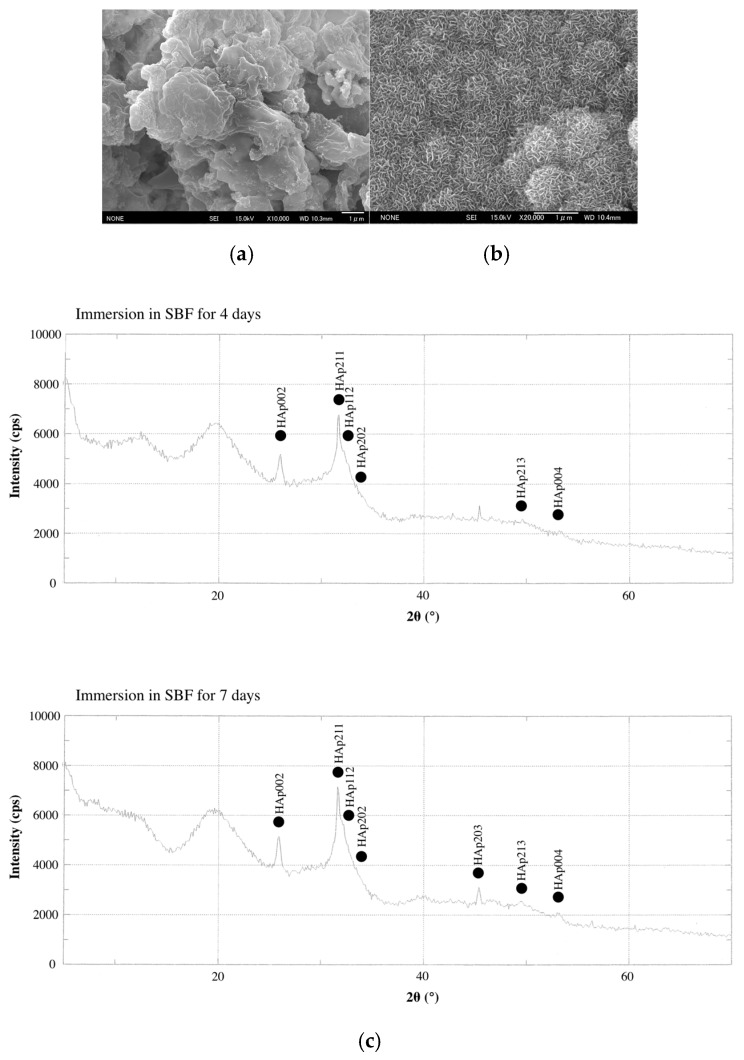

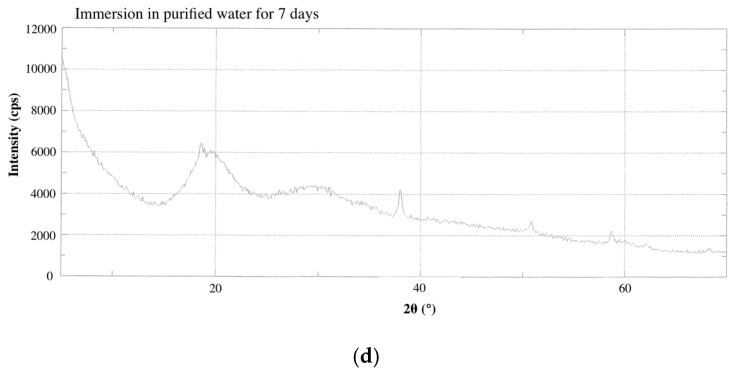
FE-SEM images and XRD patterns of the CS-BG surface. (**a**) FE-SEM images of CS-BG immersed in SBF. (**b**) FE-SEM images of CS-BG immersed in purified water. Scale bar 1 μm. (**c**) XRD patterns of CS-BG after immersion in SBF for four and seven days. (**d**) XRD patterns of CS-BG after immersion in purified water for seven days [51].

The surface-structures of CS-BG discs (diameter 3.5 mm, height 6 mm) hardened in either SBF or purified water were analyzed using Field emission scanning electron microscope analysis (FE-SEM). The surface structure of CS-BG after immersion in SBF showed typical spherules of petal-like crystals (Figure 6a). X-ray diffraction analysis (XRD) showed that petal-like crystals were HAp. In SBF, HAp crystallization on the surface increased in a time-dependent manner (Figure 6c) [51]. On the other hand, the surface structure of CS-BG after immersion in purified water showed no petal-like crystals (Figure 6b,d).

## 3. Biocompatibility

Biocompatibility is an essential property of any root filling material that is in direct contact with both hard tissues (e.g., dentin or cementum) and soft tissues (e.g., periodontal ligament) [52,53]. Reiterating, biocompatibility is the ability of a material to achieve a stable and advantageous host response during application [54]. Biocompatibility is typically assessed by cytotoxic tests in most studies [55]. The cytotoxicity of bioceramic-based sealers has been evaluated in vitro using mouse osteoblast cells, human osteoblast cells [38,56,57], and human periodontal ligament cells [58,59,60]. 

**Figure 7:**
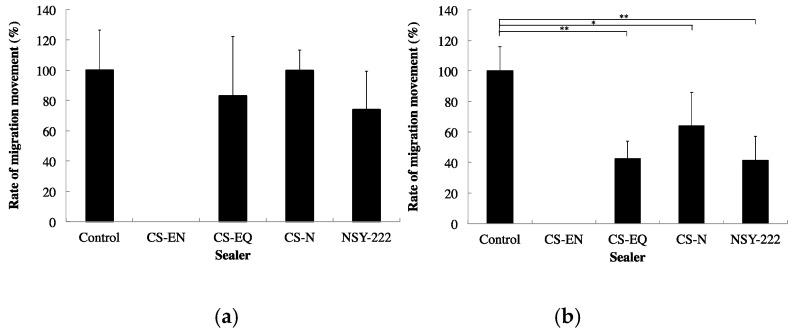
Effects of root canal filling sealers on cell migration ability. (**a**) HPDLCs. (**b**) osteoblast-like cells. The percentage of cell migration after scratching. Control: no sealer, CS-EN: eugenol-based sealer, CS-EQ: eugenol-based sealer quick, CS-N: non-eugenol-based sealer, CS-BG: BG based sealer. Each bar represents a mean ± SD. *, **: significant differences with p < 0.05 and p < 0.01, respectively [61].

**Figure 8:**
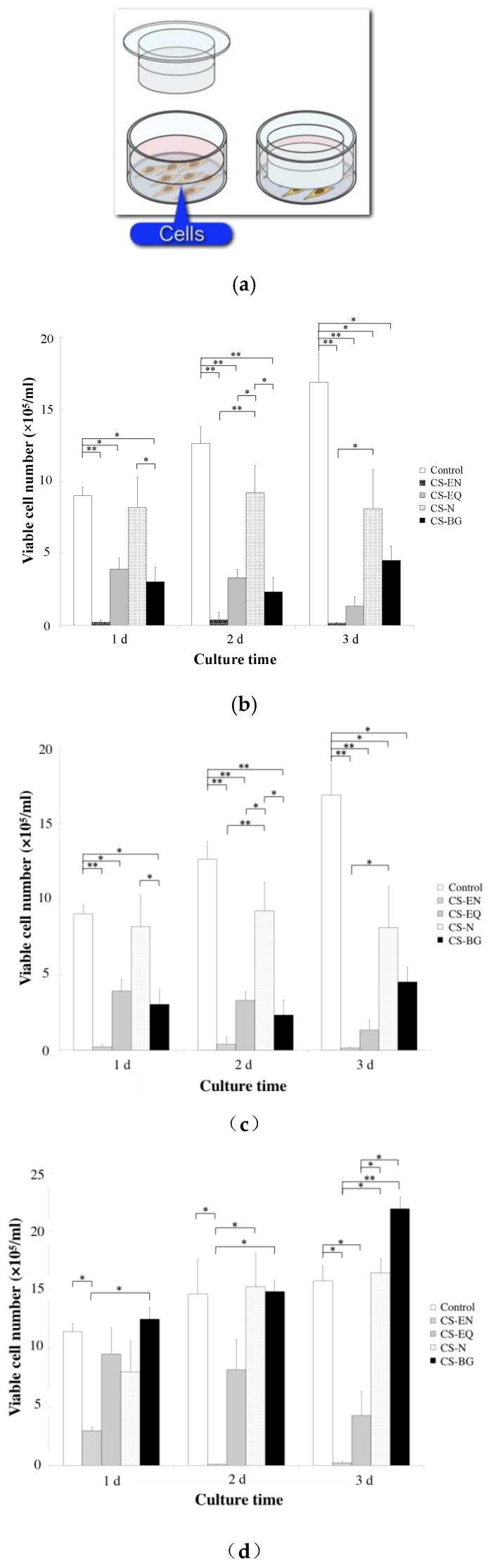

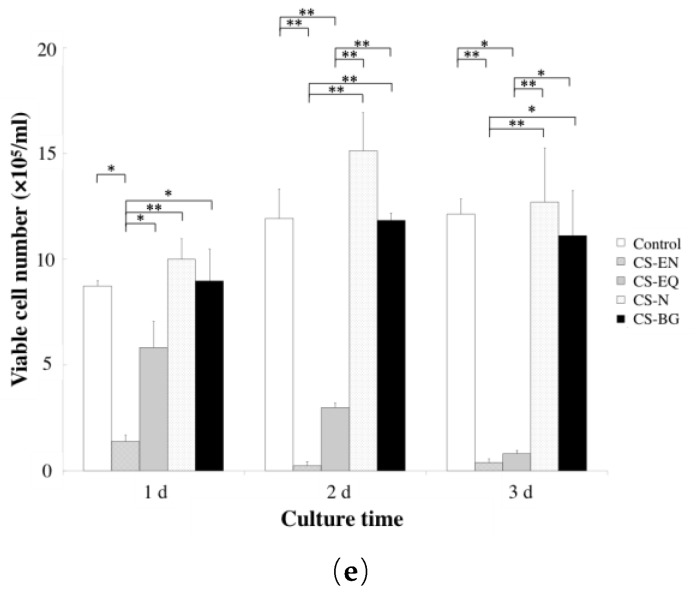
Effects of root canal filling sealers on cell viability. (**a**) Schematic of culture method. The cells (1 × 105/ well) were separately subcultured in 24-well Transwell plates. Transwell filter inserts including fresh and hardened sealers were inserted into the wells. (**b**,**c**) fresh sealer. (**d**,**e**) hardened sealer. (**b**,**d**) HPDLCs. (**c**,**e**) osteoblast-like cells. Control: no sealer, CS-EN: eugenol-based sealer, CS-EQ: eugenol-based sealer quick, CS-N: non-eugenol-based sealer, CS-BG: BG-based sealer. Each bar represents a mean ± SD. *, **: significant differences with p < 0.05 and p < 0.01, respectively [61].

**Figure 9:**
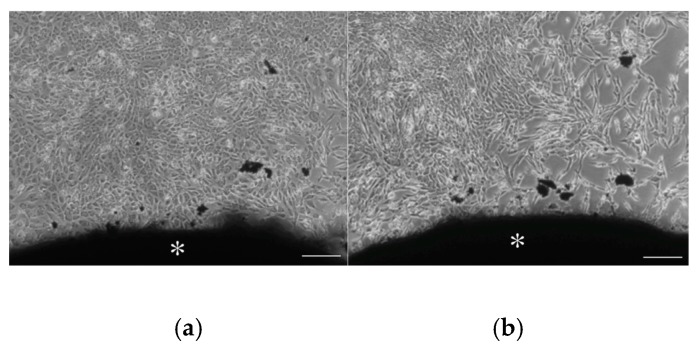
Phase-contrast microscopic photographs showed the attachment of cells to CS-BG (＊). (**a**) HPDLCs. (**b**) osteoblast-like cells [62]. Scale bar 200 μm.

**Figure 10:**
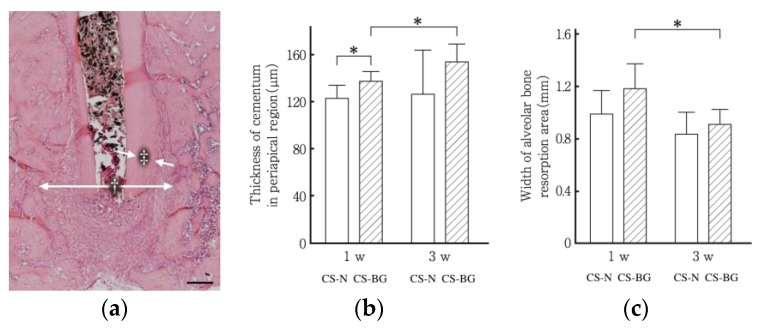
Semi-quantitative analysis of the tissue responses on periapical tissue after root canal obturation with CS-BG. (**a**) Each site of measurement for the width of periapical bone alveolar resorption (†) and the thickness of cementum (‡). Scale bar 200 μm. (**b**) Width of periapical alveolar bone resorption area. (**c**) The thickness of cementum in the periapical region. CS-N: non-eugenol-based sealer. Each bar represents a mean ± SD. *: significant differences with p < 0.05 [63].

The in vitro biocompatibility of CS-BG was demonstrated by cell migration and viability assays using human periodontal ligament cells (HPDLC) and osteoblast-like cells. Migration and survival of both HPDLC and osteoblast-like cells under CS-BG showed no significant difference when compared to control (Figure 7 and Figure 8) [61]. HPDLC and osteoblast-like cells proliferated and migrated in direct contact with the surface of hardened CS-BG (Figure 9) [62]. The in vivo biocompatibility of CS-BG was analyzed by both rat pulpectomy and root canal obturation models. These in vivo tests indicate that CS-BG does not inhibit the wound healing process of periapical tissue around the root apex of a canal filled with CS-BG (Figure 10) [63]. These in vitro and in vivo studies show that CS-BG has excellent biocompatibility for periapical tissue.

## 4. Sealing Ability

**Figure 11:**
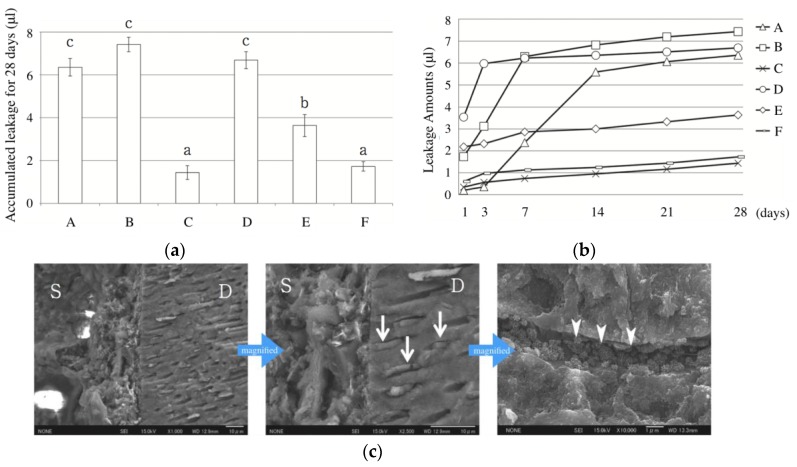
Sealing ability of root canal sealers after root canal obturation. (**a**) Time-dependent leakage evaluation of six different root canal obturations using dye penetration test. (**b**) The total leakage amount measured for 28 days. A: Eugenol-based root canal sealer (lateral condensation technique), B: Non-eugenol-based root canal sealer (lateral condensation technique), C: Bioceramics-based root canal sealer (lateral condensation technique), D: Bioceramics-based root canal sealer (single cone technique), E: CS-BG (lateral condensation technique), F: CS-BG (single cone technique). (**c**) FE-SEM images of the interface between the filled sealer and root canal wall. S: CS-BG, D: dentin. Arrows: the formation of tag-like structures in dentinal tubules, arrowheads: hydroxyapatite-like crystals in dentinal tubules [71]. Scale bar 10 μm.

The invasion of microorganisms into the interfacial region between filling materials and the root canal dentinal wall should be prevented to avoid re-infection [64,65,66]. Properly sealing this interface is dependent on the ability of the filling material to bind to the dentinal wall. There exists no standard method for measuring the sealing ability of a root canal sealer [67,68,69,70]. To assess the sealing ability of CS-BG, a dye leakage test was used to simulate the seepage of nutrient fluid into the sealed cavity. An amount of dye was sealed within the root canal and sealed with a combination of gutta-percha point and CS-BG by the lateral condensation technique. The total amount of leakage was approximately half in comparison with conventional root canal sealers (eugenol-based and non-eugenol-based sealer), and the leakage gradually decreased over time (Figure 11a,b) [71]. When a root canal was filled with CS-BG by the single-cone technique, the leakage was less than that observed for the CS-GB material applied by the lateral condensation technique (Figure 11a,b) [71]. These results showed the excellent sealing ability of CS-BG, especially when applied by the single cone method.

The characteristics of the interface between the hardened CS-BG and the root canal wall was analyzed by both FE-SEM and Energy-dispersive X-ray spectrometry (EDX). The FE-SEM showed the formation of a tag-like structure comprised of typical spherules of petal-like crystals embedded into dentinal tubules and at the entrance of the tubules (Figure 11c) [71]. These crystals were identified as HAp by EDX analysis [71]. 

**Figure 12:**
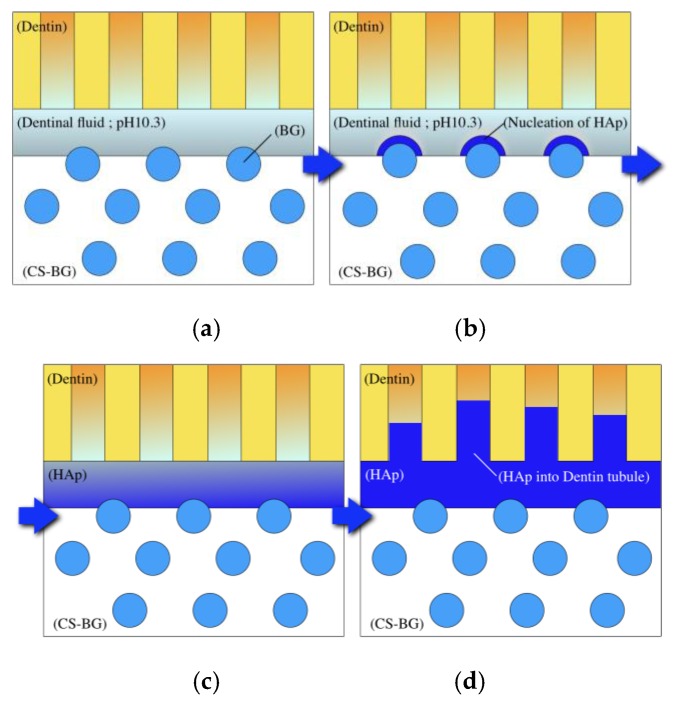
Schematic of the proposed mechanism for CS-BG to bond to dentin. (**a**) CS-BG matrix generates a pH of 10.3 in the dental fluid at the sealer-dentin interface. (**b**,**c**) CS-BG matrix displays an amphiphilic property and facilitates the growth of HAp. (**d**) After bonding with dentin, HAp crystals grow into the dentinal tubule.

Figure 12 shows a proposed mechanistic scheme for the bonding of CS-BG to the dentin-based root canal wall. After the obturation of a root canal with CS-BG, the CS-BG makes contact with a small amount of dental fluid on the dentin. During hardening, the CS-BG releases ions from its matrix, these components consist of the non-BG component of the sealing paste. The evolution of these ions maintains a pH of approximately 10 in the surrounding dentinal fluid, which is the optimal pH for the formation of HAp on the surface of BG. The BG within the sealer mixture then reacts to the dental fluid, resulting in the release of critical concentrations of soluble Si, Ca, P, and Na ions. This causes the formation of a silica-rich gel on the BG surface that reacts with the ions now present in the dentinal fluid. As a result, HAp-like crystal layers are formed on the surface of BGs. Finally, HAp-like crystal tags interstitially grow into dentin tubules. The overall CS-BG bonding with the dentin wall is formed through the formation of these HAp layers and tags within the dentin tubules (Figure 12).

## 5. Removability

**Figure 13:**
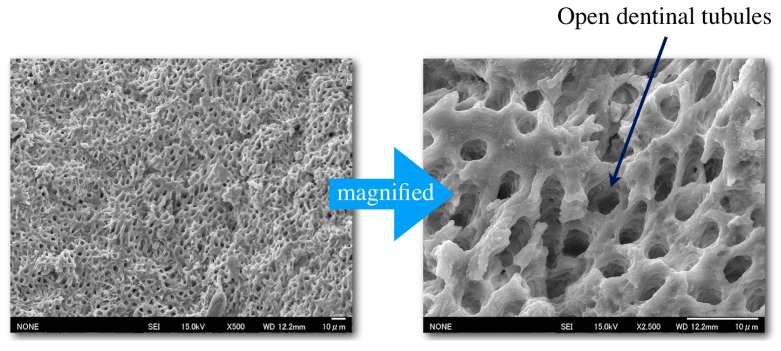
Root canal wall after the removal of hardened CS-BG and irrigation with a solution of EDTA. Dentinal tubules were observed [75]. Scale bar 10 μm.

When re-infection occurs at the periapical tissue of a treated tooth, dentists must first remove any present root canal sealer from the canal before proceeding with endodontic retreatment [72,73,74]. In vitro studies demonstrated that CS-BG is capable of being removed entirely by the standard methods of re-preparation and irrigation with an EDTA solution. Furthermore, the dentinal tubules of the dentin wall were reopened upon removal of CS-BG, shown by FE-SEM images in Figure 13 [75]. The easily removed nature of the sealer, coupled with the reopening of the dentin cavities, indicates that CS-BG does not inhibit retreatment.

## 6. Clinical Performance of Bioactive Glass-Based Root Canal Sealer

**Figure 14:**
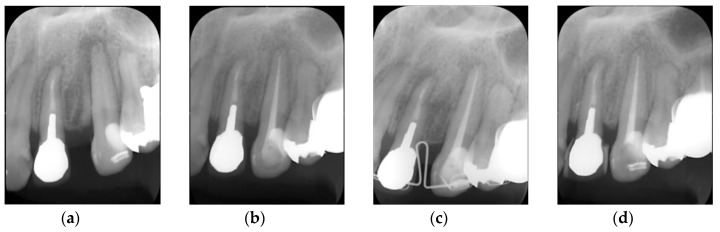
Clinical performance of CS-BG. (**a**) Pre-treatment radiograph. (**b**) Post-obturation radiograph. (**c**) 6 months follow-up radiograph. (**d**) 14 months follow-up radiograph.

CS-BG is now available for use in root canal obturation and has been shown to induce good wound healing of periapical tissues. Figure 14 shows a clinical case (40-year-old female) upon whom CS-BG was applied during a root canal obturation. The radiographic image taken during pre-endodontic treatment (Figure 14a) shows an apparent radiolucency at the periradicular area of the maxillary left canine; this radiographic translucency was diagnosed as symptomatic apical periodontitis. After standard endodontic treatment, the canal was obturated using CS-BG and gutta-percha by a non-compaction technique (Figure 14b). Wound healing and bone formation of the periapical tissues were observed at both six and 14 months after the obturation (Figure 14c,d). From now on, we will follow the cases obturated using CS-BG for longer-term clinical efficacy.

## 7. Potential of Bioactive Glass-Based Sealer as Root Canal Filling Material Without Semisolid Core Materials

At present, there are various techniques to obturate root canal systems, including sealer, sealer plus a single-core material, sealer coating combined with cold compaction of core materials, sealer coating combined with warm compaction of core materials, and sealer coating combined with a carrier-based core material [76,77]. It has also been reported that a significant number of general practitioners have chosen cold lateral or warm gutta-percha compaction with either eugenol-based or non-eugenol-based sealer [78,79,80,81,82]. However as revealed by several reviews, bioceramic-based root canal sealers applied by the single cone technique are more appropriate sealers to obtain good sealing of an obturated root canal [83,84]. When undertaking the single cone technique, the sealer delivery method is important. The root canal sealer should be delivered to the root apex before the insertion of the single-core material. Slight pressurization occurs during the insertion of core material; this pressure can cause the sealer within the canal to flow into lateral branches of the root canal. Within this review, it is indicated that CS-BG has the high sealing ability, suggestive of its potential as a sealer for root canal obturation even without semisolid core material such as a gutta-percha point. In hopes to achieve the best possible patient outcome after obturation with CS-BG—with or without a semisolid core material—the delivering device of the bioceramic-based sealer should be one of the next targets for translational research in Endodontics.

## 8. Conclusions

The next-generation bioactive glass-based root canal sealer examined in this work has displayed capabilities to form hydroxyapatite-like precipitations, biocompatibility, sealing ability, and removability. CS-BG is now available for use in root canal obturation and has been shown to induce good wound healing of periapical tissues in clinical cases. This accumulation of characteristics is suggested that CS-BG has its potential as a sealer for root canal obturation even without a semisolid core material.

## Figures and Tables

**Table 1:** Physical properties of CS-BG.

Flow	28.7 mm	Solubility	0.5%
Working time	15 mm	Disintegration	None
Setting time	180 min	Radiopacity	5 mmAl.
Film thickenss	27.9 μm		
